# Heat Shock Proteins in Tendinopathy: Novel Molecular Regulators

**DOI:** 10.1155/2012/436203

**Published:** 2012-11-05

**Authors:** Neal L. Millar, George A. C. Murrell

**Affiliations:** ^1^Institute of Infection, Immunity and Inflammation, College of Medical, Veterinary and Life Sciences, University of Glasgow, Glasgow G12 8TA, UK; ^2^Department of Orthopaedic Surgery, Orthopaedic Research Institute, University of New South Wales, St. George Hospital Campus, Kogarah, Sydney, NSW 2217, Australia

## Abstract

Tendon disorders—tendinopathies—are the primary reason for musculoskeletal consultation in primary care and account for up to 30% of rheumatological consultations. Whilst the molecular pathophysiology of tendinopathy remains difficult to interpret the disease process involving repetitive stress, and cellular load provides important mechanistic insight into the area of heat shock proteins which spans many disease processes in the autoimmune community. Heat shock proteins, also called damage-associated molecular patterns (DAMPs), are rapidly released following nonprogrammed cell death, are key effectors of the innate immune system, and critically restore homeostasis by promoting the reconstruction of the effected tissue. Our investigations have highlighted a key role for HSPs in tendion disease which may ultimately affect tissue rescue mechanisms in tendon pathology. This paper aims to provide an overview of the biology of heat shock proteins in soft tissue and how these mediators may be important regulators of inflammatory mediators and matrix regulation in tendinopathy.

## 1. Introduction

Primary disorders of tendons are common and account for a high proportion of referrals to rheumatologists and orthopaedic surgeons [1]. The most commonly involved tendons are the rotator cuff (particularly supraspinatus) in the shoulder, the forearm extensor (tennis elbow) and flexor tendons (golfers elbow) in the forearm, the patella tendon in the knee, the Achilles tendon in the lower leg, and the tibialis posterior tendon in the ankle and foot. The intrinsic pathogenetic mechanisms underlying the development of tendinopathies are largely unknown however proinflammatory cytokines, apoptosis, and mechanical stress have recently been functionally implicated in several model systems [2, 3]. Increasing evidence is emerging that repetitive tissue trauma and its associated damage in stromal tissues are recognized at the cell level via receptor-mediated detection of intracellular proteins released by necrotic cells [4]. The term “alarmin” is proposed to categorise such endogenous molecules that function to mobilise and activate immune cells after interaction with their specific receptors during host defence and tissue repair [4]. Heat shock proteins (HSPs), a type of stress molecules involved in protein folding, are implicated as important tissue alarmins [5]. HSP activation can directly affect both innate and adaptive immunity, although controversial studies and opinions exist in the field [6–8]. The innate immune responses induced by HSPs include cytokine and chemokine release and activation of NK cells [9]. Their expression in response to stress also has an important function in protection against apoptosis and in regulation of apoptotic cell signaling [10]. Thus, their evolutionary conservation and the upregulation during stress and binding to pattern recognition proteins make it logical that HSPs can act directly as danger signals in tendinopathy.

In this paper we summarize recent findings of heat shock proteins in inflammatory and tendon disease and highlight our key findings which may be important in understanding the pathogenesis of primary tendon diseases.

## 2. Heat Shock Proteins

HSPs are expressed both constitutively (cognate proteins) and under stressful conditions (inducible forms). Additionally, a variety of stressful situations including environmental, pathological, or physiological stimuli induce a marked increase in HSP synthesis, known as the stress response [11] and upon necrotic cell death, HSPs are leaked into the extracellular compartment [12]. In addition, HSPs can be released extracellularly independent of necrosis in response to a number of stressful conditions [13, 14]. However the mechanisms and physiological significance of HSP release are not clear. HSPs are present in the circulation of normal individuals [15], and their circulating levels decrease with age [16] and increase in a number of pathological conditions such as hypertension [17], atherosclerosis [18], and rheumatoid arthritis [19]. The principal HSPs range in molecular mass from ~15 to 110 kDa and are divided into groups based on both size and function [20]. They are present in the cytosol, mitochondria, endoplasmic reticulum, and nucleus, although these locations vary depending on the particular protein. The most well-studied and understood HSPs in mammals are those with molecular masses of ~60, 70, 90, and 110 kDa. These HSPs are expressed at normal body temperatures (~37°C) and in conditions of stress [21]. The primary function of the HSPs appears to serve as molecular chaperones in which they recognize and bind to nascent polypeptide chains and partially folded intermediates of proteins, preventing their aggregation and misfolding, or as molecules that directly mediate protein folding [22, 23]. Several important cytoprotective functions (the folding and unfolding of proteins, translocation of proteins across membranes, and the prevention of protein aggregation have been attributed to HSPs, in particular, the HSP70 family [24–26]. Interestingly, it has also been noted that HSPs can play a role in apoptosis with HSP27, HSP70, and HSP90 proteins predominantly antiapoptotic, while HSP60 is proapoptotic. Moreover, it appears that these HSPs function at multiple points in the apoptotic signalling pathway to elicit this response [27]. Thus their relevance to tendon disease is made all the more important due to the strong association of apoptosis in human tendon pathology [28, 29].

## 3. Heat Shock Proteins and Inflammatory Disease

Although the primary focus of research on HSPs has been directed toward their functions and accumulation inside the cell in response to a physiological stress, there is emerging recognition that HSPs serve as key modulating signals for immune and inflammatory responses [30]. One area of investigation pertinent to the topic of stress tolerance has dealt with the potential role of HSPs in cytokine production. Elevations in intracellular HSP levels have been shown to improve cell tolerance to inflammatory cytokines such as TNF-*α* and interleukin-1 [23, 31]. HSP accumulation within a cell produces both transcriptional inhibition and a decrease in TNF-*α* and interleukin-1 secretion [32]. Kluger et al. [33] demonstrated that heat conditioning and the resultant increase in intracellular HSP70 levels protected animals from an endotoxin dose that was lethal in unconditioned rats. Moreover, this paradigm was associated with a decrease in serum TNF-*α* levels after administration of endotoxin in the heat-conditioned animals [33]. These results suggest that intracellular HSP accumulation may contribute to a reduction in inflammatory cytokine production with cellular challenge.

**Figure 1:**
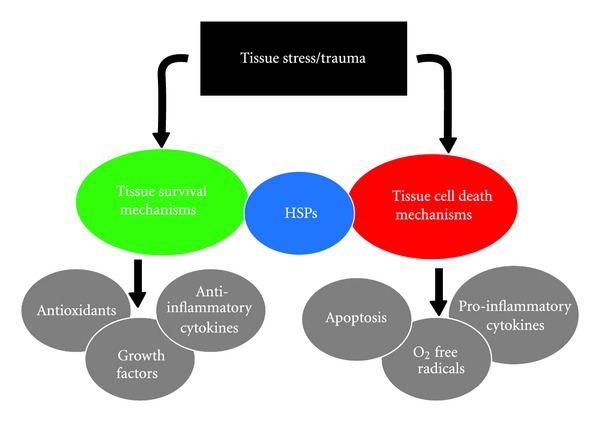
The biology of heat shock proteins in inflammatory disease. Tissue damage/stress results in the release of alarmins which in turn signal via the highlighted receptor complexes. HMGB1 and IL-33 also have intracellular nuclear functions when upregulated. This results in the release of further cytokines, growth factors, and changes in extracellular matrix production within the damaged tissue with pathological changes.

HSPs have become increasingly associated with rheumatic disease. In animal studies using Freund's adjuvant transfer of an autoreactive T cell clone recognising a determinant on the mycobacterial 65 kDa antigen was arthritogenic while prior immunisation with the 65 kDa heat shock protein abrogated this effect [34]. Human investigations have revealed that 49% of patients with ankylosing spondylitis have antibodies against HSP63 [35] while patients with systemic lupus erythematosus have serum IgG antibodies to HSP90 [36] and both IgM and IgG to HSP70 [37]. More recently various groups have highlighted elevated levels of HSPs in rheumatoid arthritis [19, 38–40] with the rheumatological community considering their merit as small molecular targets [41]. Thus HSPs released in response to tissue injury/stress seem capable of straddling the divide between tissue survival versus tissue death mechanisms in inflammatory diseases (Figure 1).

## 4. Tendinopathy

Overuse tendon injuries, namely, tendinopathies pose a significant, highly prevalent problem in musculoskeletal medicine [42] with shoulder tendon injuries alone amounting to an annual cost of $3 billion to the US healthcare system [43]. The intrinsic pathogenetic mechanisms underlying the development of tendinopathies are largely unknown however excessive cellular load and repetitive stress have been shown to be functionally important [44]. Thus the pathological process of repetitive microtrauma/stress lends itself well to the investigation of heat shock proteins which are so inextricably linked to tissue stress.

Tendinopathy is an overuse injury characterized by pain with movement, local tenderness, weakness, and decreased mobility at the injured site. These symptoms are the result of deviation from the tendon's normal physiology. In healthy tendon, 95% of tendon tissue is collagen I [45], residing within fibroblast-like tenocytes, glycoproteins, and glycosaminoglycans. Collagen III is mainly produced during tendon healing and remodelling and is biomechanically weaker then type I collagen. Macroscopically, tendons thicken and weaken in tendinopathy. Degenerative changes are found in 90% of specimens of symptomatic tendon. In addition to mucoid, hyaline, hypoxic, or fibrinoid degeneration, collagen III is observed in symptomatic tendons at a higher percentage than uninjured tendons [46]. This indicates a disruption of tissue homeostasis, specifically, excessive remodelling. Microscopically, collagen fibrils are disorganized with decreased tropocollagen cross-linking [47], glycosaminoglycan production is increased, both of which contribute to increased water retention and ultimate decrease in tensile strength (Table 1). Tenocytes become rounded and new blood vessels arise accompanied by neurogenesis. This increased neural volume is posed to cause pain in tendinopathy [48, 49].

## 5. A Human Model of Early Tendinopathy

**Figure 2:**
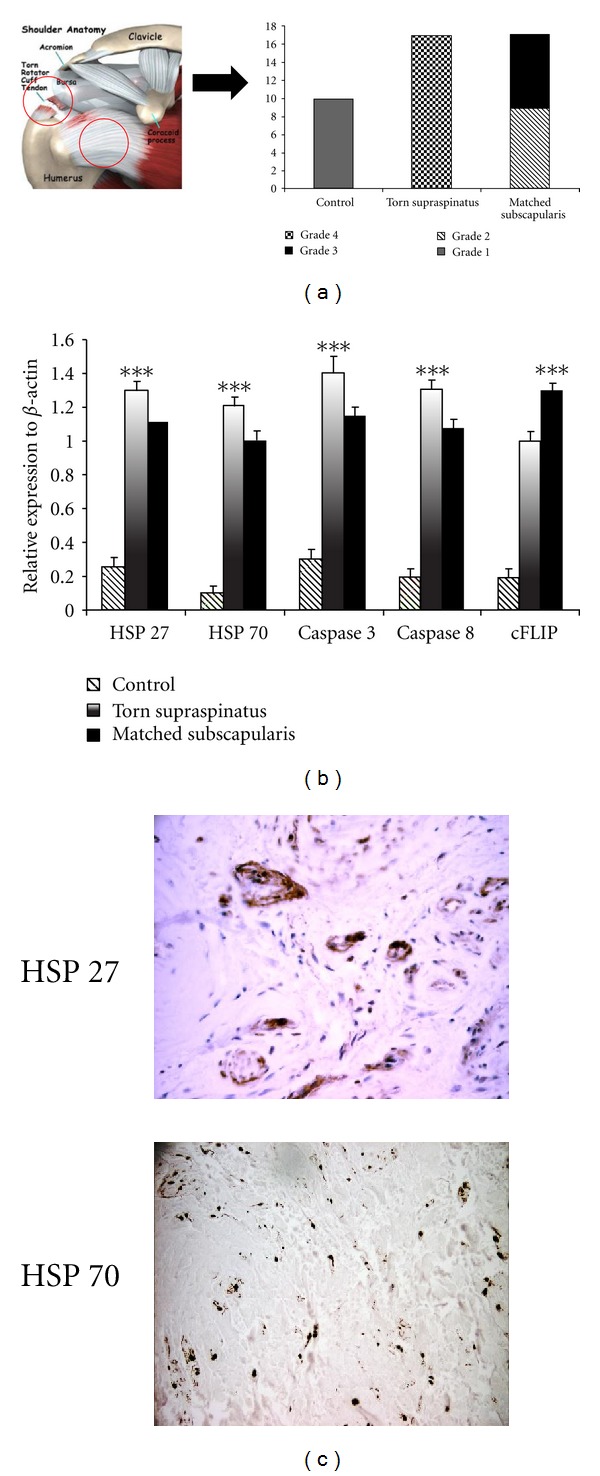
Heat shock proteins in early human tendinopathy. (a) Biopsies from subscapularis tendon revealed grade 1-2 pathological changes (mucoid degeneration, increased neovascularisation) in keeping with early tendinopathy. This was a chance finding when using this tissue as an internal control. This has subsequently allowed us to investigate alarmin molecule in early stressed tendon tissue. (b) These biopsies subsequently showed a significant increase in heat shock protein mRNA in tendon pathology. The bar graph illustrates the relative expression of apoptosis and HSP genes in human tendon samples. The data are displayed as the mean ± SEM, *n* = 17 for supraspinatus and matched subscapularis, *n* = 10 for control group (*P < 0.01; **P < 0.001). (c) The immunohistochemistry of heat shock protein HSP27 and HSP70 is shown in torn human subscapularis tendon (a) and in torn human supraspinatus tendon (b) (magnification ×200).

One of the major limitations of human studies is that tendon biopsies are usually obtained when patients are symptomatic and therefore biopsy material likely represents chronic, rather than early phase disease [50]. Medical intervention at this early stage may offer considerable therapeutic advantage over later surgical approaches. We previously demonstrated that matched subscapularis tendon from patients with full thickness rotator cuff tears represents a model of early human tendinopathy [51] based on histological appearances and significantly increased levels of cytokines and apoptotic markers in these tissues (Figure 2(a)). These studies established a human model of early tendinopathy for the first time and have been confirmed by an independent group [52]. This model has now not only allowed us to elucidate a role for HSPs in tendinopathy but also has finally allowed the targeted mechanistic investigation into key molecular events in early tendon disease [53]. 

## 6. Heat Shock Proteins in Tendon

The investigation of HSPs in tendon remains limited. Animal investigations have provided helpful insight. Pan and Halper [54] described the effects of increased temperature, mechanical stress, and growth factors on Hsp47 and type I procollagen expression in embryonic chicken tendon cells. Their data showed that transforming growth factor *β*1 (TGF-*β*1) was a key regulator of HSP47 expression as the addition of TGF-*β*1 led to a moderate increase in the expression of HSP47 mRNA. They also reported that mechanical stress increased HSP47 mRNA expression and Hsp47 protein synthesis. Induction of HSP47 protein expression by heat shock, mechanical stress and TGF-*β*1 was likely achieved through activation and translocation of heat shock transcription factor 1 into the nucleus.

Jagodzinski et al. [55] examined the expression of HSP72 in tendon fibroblasts subjected to mechanical stress. They showed that HSP72 accumulates in the nucleus with an associated transient upregulation after cyclic longitudinal stretching suggesting a role as a tissue repair mechanism. Barkhausen et al. [56] investigated the influence of repetitive cyclic longitudinal stress patterns on proliferation, apoptosis, and expression of HSP72 in tenocytes. Stress patterns applied during two days resulted in a reduced proliferation and apoptosis rate whereas the expression of HSP72 showed a significant increase. This study suggested that inhibition of proliferation and apoptosis occurred through increased HSP72 activity and may implicate it in tendon tissue reparation and tissue engineering.

Based on reports of excessive apoptosis in torn supraspinatus tendon and mechanically loaded tendon cells, we hypothesized heat shock proteins may be present in rodent and human models of tendinopathy due to their central role in caspase-dependent apoptotic cell signaling. We utilized a running rat supraspinatus tendinopathy overuse model with custom microarrays to investigate the process at a genetic level [57]. Additionally torn supraspinatus tendon and matched intact subscapularis tendon samples (“early” pathology) were collected from patients undergoing arthroscopic shoulder surgery. Overall, 91 genes were found to be significantly upregulated, and 37 significantly downregulated. The differential expression of apoptotic-related genes represented 6% (5 genes) of the significantly upregulated genes and 8% (3 genes) of significantly downregulated genes. Upregulation (*P* < 0.01) of HSP27 (×3.4) and 70 (×2.5), cFLIP (×2.2) receptor and caspase 8 (×3.1) occurred in degenerative rat supraspinatus tendon subjected to daily treadmill running for 4weeks. We further confirmed increased levels of heat shock protein and apoptotic regulatory genes in human supraspinatus and subscapularis tendon at the RNA and protein level [58] (Figures 2(b) and 2(c)). Overexpression of HSP27 is essential in preventing cells from undergoing apoptosis, a switch that may be redox-regulated [59]. HSP27 inhibits specifically the cytochrome C and ATP-triggered activity of caspase 9 on the apoptotic pathway. Furthermore, HSP27 indirectly interferes with cell death because of its ability to modulate intracellular glutathione [60], a parameter that is also regulated by exercise. Cytochrome C also triggers the oligomerization of Apaf-1, which in turn recruits pro-caspase 9 and pro-caspase 3 into the apoptosome (the caspase activation multiprotein complex). HSP70 interacts with Apaf-1 thereby preventing its interaction with the caspases preventing apoptosis. HSP70 also protects cells from heat stress [61], from the cytotoxic effects of TNF*α* [62], and from nitric oxide [63]. Based on these observations it would appear that heat shock proteins act as a check rein to apoptotic cell damage in tendinopathy. 

## 7. Summary

**Figure 3:**
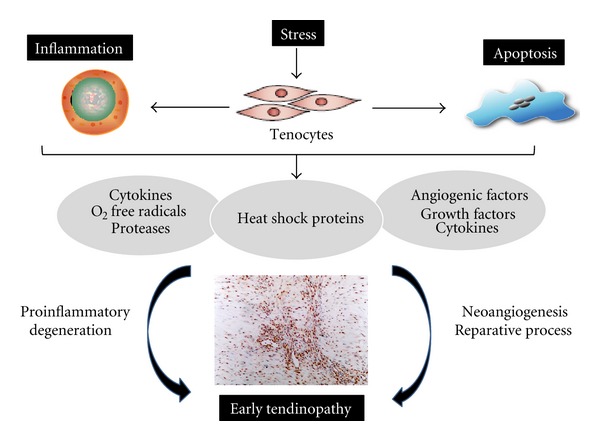
Overview of heat shock biology in tendinopathy. Schematic diagram illustrating the manner in which early tendinopathy may arise due to HSP release. An increase in stress that a tendon cell experiences results in the release of various inflammatory mediators and associated HSPs that interact to drive the tendon matrix toward a degenerative or reparative process.

In conclusion, heat shock proteins are components of nature's immune response that can act in a positive or negative way to the host immune system during the course of disease. These molecules seem to act as early regulators of the decision of a tissue/cell towards a reparative versus degenerative/inflammatory pathological process in joint related diseases. Repetitive microtrauma/stresses are now considered as one of the main pathophysiological causes of tendinopathy. Our investigations in early tendon damage have revealed a role for a heat shock proteins along with other investigators. We propose that when these molecules are released from stressed tenocytes they act as orchestrators of both the tissue healing response and subsequent inflammatory reaction with a fine balance between reparative versus degenerative change (Figure 3). Further work is ongoing within our institute to further elucidate their mechanistic role and possible therapeutic targeting.

## Figures and Tables

**Table 1:** Key pathological features of tendinopathy.

Findings	Macroscopic	Light microscopy	Ultrasound findings
Normal tendon	(i) Brilliant white (ii) Firm Fibroelastic texture	(i) Organised parallel collagen bundles (ii) Spindle-shaped tenocyte nuclei (iii) Parallel nuclei alignment	(i) Regular uniform fibre structure (ii) Parallel hyperechoic features

Tendinopathy	(i) Grey or brown (ii) Thin tissue, fragile, and disorganised (iii) Loose texture	(i) Disorganised collagen bundles (ii) Round dark stained tenocyte nuclei (iii) Increased number of nuclei with loss of parallel arrangement (iv) Mucoid degeneration and vacuoles (v) Increase of vascular and nerve ingrowth (vi) Increased ground substance and GAG	(i) Local hypoechoic areas (ii) Irregular fibre structure (iii) Neovascularisation on power doppler (iv) Widening of tendon
